# Late onset and early onset aura: the same disorder

**DOI:** 10.1007/s10194-012-0419-8

**Published:** 2012-02-17

**Authors:** Isabel Pavão Martins, Tomás Goucha, Ines Mares, Ana Filipa Antunes

**Affiliations:** 1 Department of Clinical Neurosciences (UNIC), Lisbon Faculty of Medicine, Instituto de Medicina Molecular (IMM), Hospital de Sta Maria, 1600 Lisbon, Portugal; 2 Max Planck Intitute for Human Cognitive and Brain Sciences, Leipzig, Germany; 3 GAPIC, Lisbon Faculty of Medicine, Lisbon, Portugal

## Abstract

Late onset aura (LOA) is usually considered benign but raises diagnostic uncertainties. We compared individuals with LOA (>45 years of age at aura onset) with those of early onset (EOA) in clinical features, vascular risk factors and imaging, in a retrospective study design including patients with migraine aura and age >44 years at first visit. In 77 cases (51 EOA and 26 LOA), no differences were found in gender distribution, family or personal history of migraine without aura, type of aura symptoms or imaging findings. LOA patients’ were more likely to not fulfil all ICHD-II aura criteria and to lack headache. This data suggest that LOA and EOA are overall identical but there are differences in presentation that deserve a better characterization by a prospective study.

## Introduction

“Late life migraine accompaniments” were first described by Fisher in individuals whose aura began after the 40th decade [1]. Although there is an evidence supporting the benign nature of this syndrome [2], it raises diagnostic doubts, particularly because subjects with aura are more likely to suffer silent brain infarcts [3], epilepsy [4] and all-cause mortality, including cardiovascular disease [5]. Thus, it is relevant to know if auras of late onset have any particular features or complications compared to their typical counterparts.

## Methods

We conducted a retrospective analysis of the last 100 patients observed in a headache outpatient clinic with (a) age ≥45 years at first visit and, (b) the clinical diagnosis of migraine aura. All types of aura were included, namely auras without headache, with non-typical or typical migraine headache or persistent auras, as defined by the ICHD-II [6].

Patients were divided into two groups: early onset aura (EOA), if aura began before their 45th anniversary and late onset aura (LOA) if later than that. This age cut-off was mentioned by Fisher [1] and it represents a turning point in migraine epidemiology, with a decline in prevalence and incidence [7]. Besides, it has been used as a cut-off point for stroke in young adults [8]. To ensure that both groups were comparable in age, we only included subjects with a minimum age of 45 at first visit, independently from their follow-up time in clinic or if they had migraine beginning years before. However, to avoid recall bias, data collected concerned only symptoms recorded at first and follow up consultations and not their past symptoms.

Clinical evaluation followed a semi-structured interview, neurological examination and record of diagnosis and exams performed. Data collected from records comprised: age at first consultation and of aura onset, gender, type of aura symptoms, clinical diagnosis, ICHD-II criteria [6], frequency of aura attacks including what we designated as “aura status” (i.e., the recurrence of aura episodes in a short period of time, with a minimum of three in 24 h), vascular risk factors, neurological co-morbidities (namely stroke, epilepsy, episodes of vertigo, depressive symptoms and sleep complaints) and results of neuroimaging and EEG. Comparisons were calculated using non-parametric tests for categorical variables and independent samples *t* tests for continuous variables (significance level of α = 0.05). The project was approved by the Ethics Committee of the Faculty of Medicine.

## Results

In 100 identified cases, five did not fulfil inclusion criteria and records were unavailable in 18, producing a sample of 77 case records. Main findings are summarized in Table 1. No differences were found in gender distribution, type of aura symptoms, and frequency of migraine without aura or family history of migraine. Patients with LOA were more likely to have attacks of aura without typical migraine headache compared to EOA. Aura’s symptoms were mostly visual. Aura duration, divided in <20, 20–60 and >60 min, was identical in both groups (respectively 23.3, 56.7 and 20% in EOA and 47.8, 39.1 and 13% in LOA, *p* = n.s.). Monthly frequency of aura attacks was also similar (3.4 ± 6.6 in EOA and 3.8 ± 6.3 in LOA, *p* = n.s.), as was the occurrence of “aura status”, as previously defined. There were more cases in LOA with the diagnosis of probable migraine aura (ICHD-II) than among EOA. Reasons were the presence of a single aura episode (*N* = 5) and a longer duration >60 min (*N* = 3).

**Table 1:** Population characteristics and clinical features by study group

	Total	Early onset aura (EOA)	Late onset aura (LOA)	Statistics: Chi-Square, Fisher exact test or *t* test
*N*	77	51	26	
Gender (F:M)	59:18	40:11	19:7	n.s.
Age at consultation (mean ± SD)	53.6 ± 7.8	50.5 ± 5.6	59.6 ± 8.2	*t* = −5.1,* p* < 0.001
Age at aura onset (mean ± SD)	36.6 ± 19.1	25 ± 12.5	55.2 ± 11.6	*t* = −9.8, *p*< 0.001
Clinical diagnosis (AWMH:AwoMtH)	55:22	45:6	10:16	χ^2^(1) = 20.9, *p* < 0.001
Attacks of MWoA (Y:N)	48:29	31:20	17:9	n.s.
Attacks of AWnMH (Y:N)	11:66	9: 42	2:24	n.s.
Attacks of AWoMH (Y:N)	33:44	15:36	18:8	χ^2^(1) = 11.1, *p* = 0.001
“Aura status” (Y:N)	8:68	4:47	4:21	n.s.
Aura symptoms				
Visual (Y:N)	74:3	50:1	24:2	n.s.
Only negative	11/65 (16.9%)	7/42 (16.7%)	4/23 (17.4%)	n.s.
Only positive	15/65 (23%)	10/42 (23.8%)	5/23 (21.7%)	
Positive and negative symptoms	39/65 (60%)	25/42 (59.5%)	14/23 (60.9%)	
Somatosensory (Y:N)	19:58	14:37	5:21	n.s.
Aphasia (Y:N)	21:56	12:39	9:17	n.s.
Other (Y:N)	7:70	5:46	2:24	n.s.
Multiple (Y:N)	22:54	13:38	9:16	n.s.
Aura fulfilling ICHD-II criteria (Y:N)	49:11	32 (91.4%):3	17 (68%):8	Fisher exact test, *p* < 0.05
Family history of migraine (Y:N)	43:24	28:15	15:9	n.s.
Follow-up time (years)	2.6 ± 4	2.3 ± 3.7	3.4 ± 4.5	*p* = n.s.
Co-morbidities				
Hypertension (Y:N)	24 (32.9%):49	11 (23.4%):36	13 (50%):13	χ^2^(1) = 5.36, *p* < 0.02
Diabetes mellitus (Y:N)	1:72	1:47	0:25	n.s.
Other cardiovascular disease (Y:N)	6:61	4:39	2:22	n.s.
Depressive symptoms	25/66 (37.8%)	13/44 (29.5%)	12/22 (54.5%)	χ^2^(1) = 3.89, *p* < 0.05
Sleep complaints (Y:N)	17:49	11:33	6:16	n.s.
Seizures (Y:N)	4:51	4:38	0:13	Fisher exact test, *p* = n.s.
Stroke (Y:N)	2:68	2:46	0:22	n.s.
Vertigo (Y:N)	18:38	10:24	8:14	n.s.
Normal imaging (Y:N)	44:18	29:13	15:5	n.s.
WMH (Y:N)	8:54	5:37	3:17	n.s.
Vascular lesions (Y:N)	6:56	4:38	2:18	n.s.
Non-vascular lesions (Y:N)	9:53	6:36	3:17	n.s.
Normal EEG (Y:N)	14:5	8:5	6:0	n.s.

*F* females, *M* males, *AWMH* typical aura with migraine headache, *AWnMH* typical aura with non-migraine headache, *AwoMH* typical aura without migraine headache, *AwoMtH* aura without migraine-type headache (AWnMH + AwoMH), *MWoA* migraine without aura, *WMH* white matter hyperintensities

Most patients (78%) had brain imaging and the majority were normal, with no group differences in the type of abnormalities found (white matter hyperintensities, vascular or non-vascular lesions). Although two EOA cases had abnormalities that may underlie symptomatic headache (tumour and arteriovenous malformation), those had no temporal relation with migraine, making causality unlikely.

## Discussion

The late onset of migraine aura is often a source of concern and clinicians must consider other causes of transient neurological symptoms such as TIAs, epilepsy or ophthalmological disorders.

The present study aimed to understand if patients with LOA shared clinical features, risk factors and co-morbidities with EOA. The results were overall identical, suggesting that we stand before a single condition, i.e. migraine. In fact, the two groups had a predominance of females and reported identical family and personal history of migraine headaches, thus, sharing the same gender and genetic susceptibility. Furthermore, there were no differences in imaging findings or neurological co-morbidities except that LOA patients were more likely to have hypertension and depressive symptoms, which may simply result from their higher age, hence not allowing inferences of causality. Although sleep disturbances may trigger attacks, they were similar in both groups.

Concerning the clinical diagnosis, LOA patients often presented with isolated auras, auras with atypical headache, or only fulfilled ICHD-II criteria of probable migraine with aura, whereas EOA patients presented mainly aura with migraine headache. This is in accordance with the studies demonstrating phenotype changes in migraine in lifetime with decreasing headache and associated symptoms [7]. Identical findings were reported in the Framingham study [2] (only 19% of participants fulfilled ICHD-II criteria) and in Fisher’s study [1] (27% subjects had a single attack and 27% prolonged attacks, similarly to our study).

Aura characteristics, including type, duration and frequency, were similar in both groups. An analysis of the same data with an age cut-off at 65 years yielded identical results, suggesting an age-related trend in migraine phenotypic presentation with less headache and more aura phenomena, which so far has not been explained.

We acknowledge limitations to this study, the most important being its retrospective design. Lack of systematic record of all variables produces biases favouring positive findings. In addition, the inclusion of patients older than 44 years at first visit may bias subjects towards those with late migraine aggravation, but this is balanced by the presence of a group matched by “late” consultation.

In summary, this study supports the essentially benign nature of LOA but also underlines some of its particular features. Since it does not allow firm conclusions, we highlight the need to conduct a further study with a longitudinal prospective design.
